# Supplementary material for: KOnezumi-AID: Automation Software for Efficient Multiplex Gene Knockout Using Target-AID
Source: Int J Mol Sci. 2024 Dec 17;25(24):13500. doi: 10.3390/ijms252413500 (PMC11679502; doi:10.3390/ijms252413500)
Supplement: Supplementary file 1 [file ijms-25-13500-s001.zip › ijms-3331625-supplementary.pdf]

Supplementary Figure

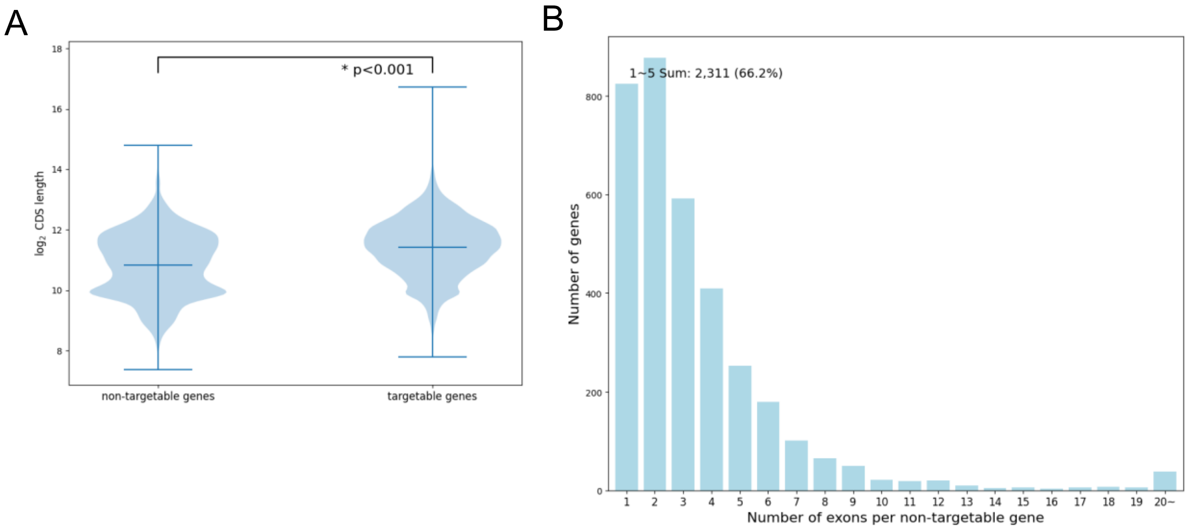

Figure S1. Non-targetable gene analysis. (**A**) Violin plot shows the distribution of the log2 coding sequence (CDS) lengths of genes with at least one candidate guide RNA (gRNA; targetable genes) and those with no candidate gRNA (non-targetable genes). Solid lines in the middle of each violin indicate the mean CDS length for each group, whereas the top and bottom edges indicate the range of the data. (**B**) Number of exons per non-targetable gene. Distribution of the number of exons for genes with no candidate gRNAs is shown. Number of exons is capped at 20 for visualization. Genes with 1–5 exons are grouped, and their total sum and percentage relative to the number of non-targetable genes are shown.

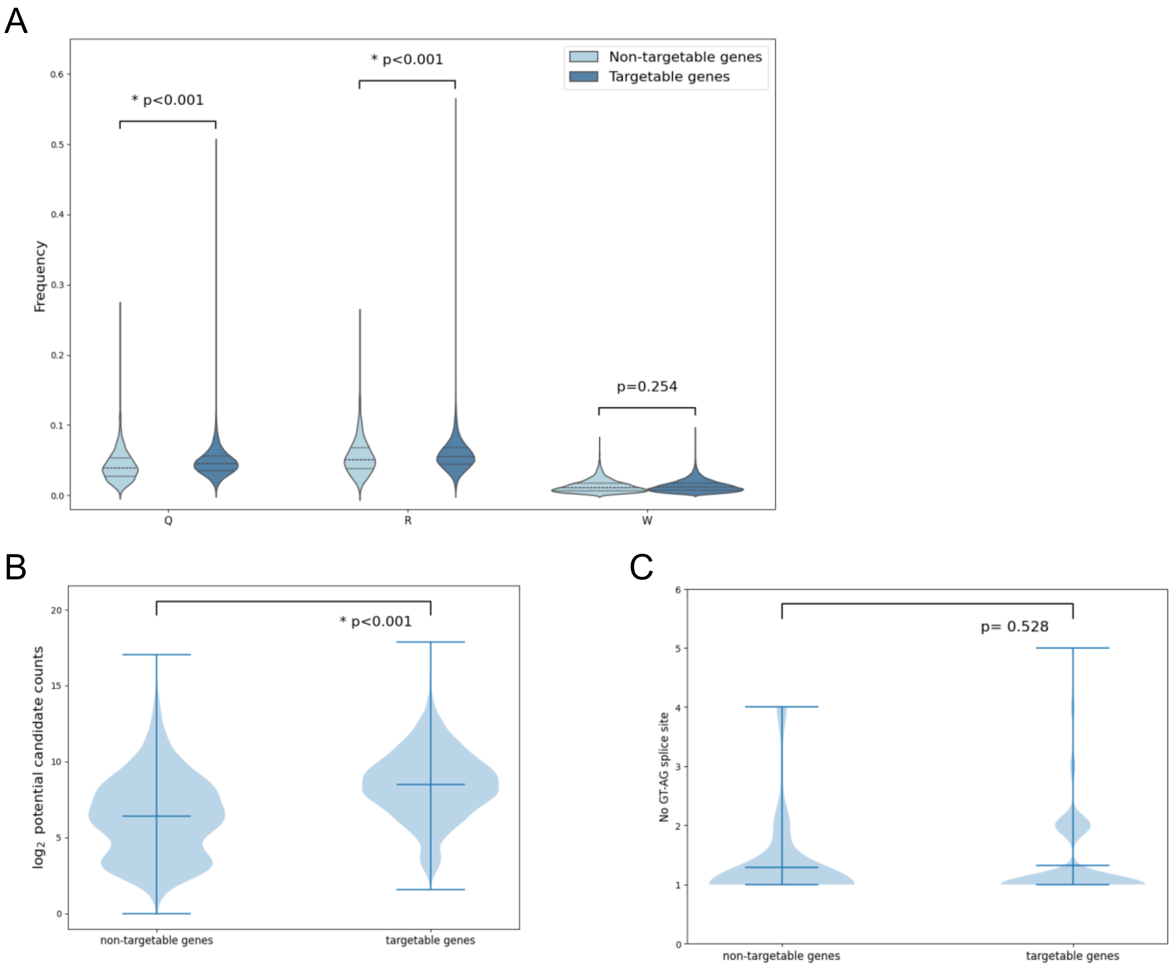

Figure S2. Characteristics of non-targetable genes in mice. (**A**) Violin plot shows the relative frequencies of amino acids that Target-AID can potentially target for inducing premature termination codon in non-targetable genes (light blue) and targetable genes (steel blue). R, Q, and W represent arginine, glutamine, and tryptophan, respectively. Dotted lines indicate quartiles. (**B**) Violin plot displays the distribution of log2 counts for sequences containing C within the target-window for non-targetable and targetable genes. The solid line indicates the mean count, with the range represented by the top and bottom edges of the violins. (**C**) Distribution of splice sites deviating from the GT-AG rule in non-targetable and targetable genes. For visualization, zeros were excluded and values of five or higher are capped at five. The solid line marks the mean count, with the range depicted by the edges of the violins.

Table S1. Comparison of execution times (in seconds) between batch processing and the cumulative execution times of individual gene analyses.

Sample_counts	Run_type	Epoch_1	Epoch_2	Epoch_3	Epoch_4	Epoch_5	Epoch_6	Epoch_7	Epoch_8	Epoch_9	Epoch_10
10	Batch	12.1171	12.0794	12.2630	12.4365	14.2786	12.8197	12.5637	12.2586	12.5610	12.5280
	Single	17.0377	17.0632	16.8153	16.9781	17.3015	17.3504	17.0478	16.8733	17.0169	17.0941
50	Batch	61.8689	60.0767	60.4172	59.8115	61.1467	60.4642	61.2221	60.5359	60.3967	60.4165
	Single	84.8209	84.3182	84.8189	84.4683	85.8179	84.7925	86.1357	84.8511	84.5561	85.1992
100	Batch	120.0737	121.0901	121.0877	119.7662	119.6276	118.8338	120.2280	119.3158	119.3670	119.2708
	Single	169.6407	169.6435	169.8945	169.5172	170.4430	169.7088	170.7648	169.6922	169.5586	169.7652
500	Batch	605.3900	604.1769	604.6586	603.8855	602.9309	603.0654	604.1634	605.0248	603.8893	603.2138
	Single	850.8517	849.6411	850.2314	849.7411	848.0108	849.4096	849.5223	850.7635	850.1582	849.2134
1000	Batch	1201.8060	1203.4392	1201.3022	1202.5259	1203.8694	1206.0710	1209.7012	1210.5353	1208.0546	1209.0075
	Single	1699.0604	1699.7841	1697.7514	1699.5501	1697.1434	1699.6249	1701.6548	1700.2637	1700.6083	1699.6944
